# Joint quantile disease mapping with application to malaria and G6PD deficiency

**DOI:** 10.1098/rsos.230851

**Published:** 2024-01-03

**Authors:** Hanan Alahmadi, Janet van Niekerk, Tullia Padellini, Håvard Rue

**Affiliations:** ^1^ Statistics Program, Computer, Electrical and Mathematical Sciences and Engineering Division, King Abdullah University of Science and Technology (KAUST), Thuwal 23955-6900, Makkah, Kingdom of Saudi Arabia; ^2^ Statistics and Operations Research Department, King Saud University (KSU), Riyadh 11564, Riyadh, Kingdom of Saudi Arabia; ^3^ Department of Epidemiology and Biostatistics, Imperial College London, London, UK

**Keywords:** Bayesian analysis, disease mapping, integrated nested Laplace approximation, joint quantile regression

## Abstract

Statistical analysis based on quantile methods is more comprehensive, flexible and less sensitive to outliers when compared to mean methods. Joint disease mapping is useful for inferring correlation between different diseases. Most studies investigate this link through multiple correlated mean regressions. We propose a joint quantile regression framework for multiple diseases where different quantile levels can be considered. We are motivated by the theorized link between the presence of malaria and the gene deficiency G6PD, where medical scientists have anecdotally discovered a possible link between high levels of G6PD and lower than expected levels of malaria initially pointing towards the occurrence of G6PD inhibiting the occurrence of malaria. Thus, the need for flexible joint quantile regression in a disease mapping framework arises. Our model can be used for linear and nonlinear effects of covariates by stochastic splines since we define it as a latent Gaussian model. We perform Bayesian inference using the R integrated nested Laplace approximation, suitable even for large datasets. Finally, we illustrate the model’s applicability by considering data from 21 countries, although better data are needed to prove a significant relationship. The proposed methodology offers a framework for future studies of interrelated disease phenomena.

## 1. Introduction

Malaria is considered a leading cause of mortality worldwide, and the disease is most prominent in Africa. It has been estimated that malaria affected about 219 million people and caused around 435 000 deaths in 2017 [1]. The Malaria Atlas Project [2] provides a global database on malaria risk in order to solve critical questions. This project disseminates free, accurate and up-to-date geographical information on malaria and associated topics. One of its research outputs points out a relationship between malaria and glucose 6 phosphate dehydrogenase (G6PD) deficiency, a genetic disorder that affects red blood cells. The G6PD is a gene that provides instructions for making the glucose-6-phosphate dehydrogenase enzyme. The research by the Malaria Atlas Project found that G6PD deficiency is common in populations that have a high level of malaria infection [3]. Studies dating back to the early 1960s [4,5] postulated that G6PD deficiency inhibits the occurrence of malaria. The reasoning was that G6PD deficiency leads to the accumulation of oxygen radicals inside red blood cells (H_2_O_2_). This accumulation offers resistance against malaria infection because the *Plasmodium falciparum* parasite (the parasite that causes malaria) does not have any antioxidant mechanism, which makes it more vulnerable to oxygen radicals [6,7]. The hypothesis that G6PD deficiency provides some protection against *P. falciparum* malaria was further supported by a review by Greene [8], based on experimental and population studies. At the same time, it was acknowledged that there are not enough data in population studies, due to limited sample sizes, to produce concluding evidence [8,9]. However, there are opposing arguments, also based on limited population studies, stating that G6PD deficiency by itself is unlikely to produce a significant protection against malaria (see [10]). In 1995, Ruwende *et al.* [11] suggested, from two case-control studies of more than 2000 African children, that G6PD deficiency reduced the risk of severe malaria by around 50%. In 2017, a systematic review by Mbanefo *et al.* [12] based on a selection of 28 various studies arrived at the conclusion that G6PD deficiency could potentially offer some protection against uncomplicated malaria, but less likely so for severe malaria.

Following the results of Allison & Clyde [5] and Beutler [9], it is of interest to perform a statistical inference of such a relationship between diseases and quantify the uncertainties involved. A joint mean regression disease mapping model could provide insights into how the mean risk of malaria is correlated with the mean risk of G6PD. However, to analyse the anecdotal evidence regarding the hypothesized link between the diseases we need to investigate the correlation between different risk quantiles instead of the means. We thus need a joint quantile disease mapping approach where different quantiles can be considered. In the framework of disease mapping, the number of cases is a discrete random variable most often modelled as a Poisson random variable where the risk is modelled instead of the actual count by including an offset for the exposure. Multivariate (or joint) disease mapping provides insights as to the risk of multiple diseases and their correlation with each other on the mean level and is described in detail by Martínez-Beneito & Botella-Rocamora [13]. As far as the authors are aware, there is no available literature on joint quantile disease mapping, to which we aim to contribute in this study.

Quantile regression was introduced by Koenker & Bassett [14]. Since then quantile regression has been widely used, also in Bayesian spatial analysis by Reich *et al.* [15]. Moreover, spatial quantile regression is widely used with other applications ranging from modelling of wildfire risk [16] to studying healthy life years expectancy [17] to economics [18]. In most works, however, the response variable is assumed to be continuously distributed and the asymmetric Laplace distribution (ALD) likelihood [19] is used to model the quantiles, irrespective of the data-generating distribution. This provides a non-parametric approach for quantile regression which seems feasible in most cases. However, the ALD approach inherently assumes that the data are continuous. In the case of disease mapping, the data are always discrete. A naive application of the ALD to discrete data often results in quantile crossing, since the resulting interpolation does not respect the discreteness of the data. Various works have been proposed that suggest different approaches to smooth and interpolate the data to a certain degree such as jittering by Machado & Silva [20] and density regression by Canale & Dunson [21]. Recently, Liu *et al.* [22] proposed a quantile regression model for discrete data by developing a discrete version of the ALD likelihood function. These approaches however cannot be applied to disease mapping directly due to the exposure difference between observational units that should be incorporated into the model directly. Even though quantile regression is often used to circumvent parametric assumptions and restrictions, in disease mapping the parametric model is necessary and should be respected by the quantile regression model. Model-based quantile regression has been used by Chambers *et al.* [23] to develop a negative binomial regression *α*-quantiles approach with an ecological regression model for application to disease mapping of lip cancer.

We propose a model-based joint quantile disease mapping model where disease counts are assumed to follow a Poisson distribution with appropriate exposure levels, and the quantiles of the risks are modelled jointly. The likelihood is constructed from a continuous approximation of the Poisson likelihood enabling the linking of the quantile regression model to the canonical parameter of the likelihood. We perform the Bayesian inference of this model using the integrated nested Laplace approximation (INLA) approach [24] as to put forth a practical, efficient and accurate framework for joint quantile disease mapping to practitioners. Most Bayesian approaches are based on Markov chain Monte Carlo (MCMC) methods but they suffer from convergence issues and/or impractical computation times. INLA has been shown to achieve the accuracy of MCMC methods with much less computational cost (in terms of time and memory requirements) [24–26].

Disease mapping and some details are presented in §2 where we include the case of joint disease mapping through multiple correlated mean regressions. Then we introduce the model-based quantile regression model for counts in §3 and show some properties with simulation studies. After extending model-based quantile regression model for counts to the disease mapping framework for one disease in §3, the joint quantile disease mapping model is proposed in §4 and applied to the data of malaria and G6PD deficiency cases in §5. The paper is concluded with some discussion of the contributions, shortcomings and possible future extensions in §6.

## 2. Disease mapping

### 2.1. Introduction

Disease mapping, also known as spatial epidemiology, analyses the incidence of disease using geographical information thus describing the spatial variation of a disease. The two characteristics of disease mapping are the location of the events, which is called spatial or geographical distribution, and the disease itself. The Poisson distribution is often used to model the incidence of diseases by taking the difference in exposure of observational units into account. For a region that consists of *n* non-overlapping areas, let *y*_*i*_ denote the number of cases in region *i*. Often *y*_*i*_ is assumed to be distributed as

2.1 $$y_{i}∼\text{Poisson}(\mu_{i}),$$

where *μ*_*i*_ is the mean and the variance of *y*_*i*_. The mean function often consists of two components. The first component is usually called the relative risk, which represents the risk within a region; it is unknown and the purpose of this work to estimate these values. The second component is usually called standardization, where the different exposures are taken into account. The expected local count is the value that represents our expected incidence if the population behaved locally in a similar way as globally. The expectation of the cases in region *i* can be written as follows:

2.2 $$E(y_{i})=\mu_{i}=E_{i}\lambda_{i},$$

where *E*_*i*_ is the expected incidence for the *i*th area and *λ*_*i*_ is the relative risk for the *i*th area [27]. The expected number can be obtained by using indirect standardization, as follows:

2.3 $$E_{i}=\sum_{\;j=1}^{m}r_{\;j}^{\left(s\right)}n_{\;j}^{\left(i\right)},$$

where $n_{\;j}^{\left(i\right)}$ is the number of experimental units in stratum *j* of area *i*, *m* is the total number of strata and $r_{\;j}^{\left(s\right)}$ denotes the disease rate in stratum *j* of the standard population. The disease rates $r_{\;j}^{\left(s\right)}$ in *E*_*i*_ for each stratum *j* are not known; they are estimated from the aggregate population data as in equations (2.4), (2.5) and (2.6):

2.4 $$r_{\;j}^{\left(s\right)}=\frac{\sum_{i=1}^{n}y_{ij}}{\sum_{i=1}^{n}n_{ij}}$$

where *y*_*ij*_ is the number of cases in stratum *j* of area *i*, and *n*_*ij*_ is the number of experimental units in stratum *j* of area *i*. We can express the disease rates in another form as follows:

2.5 $$r_{\;j}^{\left(s\right)}=\frac{y_{\;j}^{\left(s\right)}}{n_{\;j}^{\left(s\right)}},$$

where $y_{\;j}^{\left(s\right)}$ is the number of cases in stratum *j* of the standard population, and $n_{\;j}^{\left(s\right)}$ is the number of experimental units in stratum *j* of the standard population. In applications where strata information is not available, the disease rates can be computed simply as

2.6 $$r^{\left(s\right)}=\frac{\sum_{i=1}^{n}y_{i}}{\sum_{i=1}^{n}n_{i}},$$

where *y*_*i*_ is the number of cases of area *i*, and *n*_*i*_ is the number of experimental units in area *i*. A discussion about the expected number for all regions, which we denote by *E*_*i*_, is necessary. The previously defined disease rates somewhat oversimplify the situation by treating *E*_*i*_ as a fixed value, even when it is reliant on our estimation of *r*^(*s*)^. However, as the number of regions *n* grows, this approximation becomes less significant. It is also worth noting that the main purpose of defining *r*^(*s*)^ is to determine the grand intercept, *m*_0_, which is not of major interest in this context [28].

Note that *λ*_*i*_ = 1 means there is no augmented or lower risk in comparison with the whole study area while *λ*_*i*_ > 1 and *λ*_*i*_ < 1 indicate higher risk and lower risk than the average, respectively. The maximum likelihood estimator of *λ*_*i*_ is $\hat{\lambda_{i}}=y_{i}/E_{i}$, which corresponds to the standardized mortality ratio (SMR). However, mapping SMRs directly is misleading and insufficient for regions with small populations [28]. Therefore, the covariates need to be incorporated in order to smooth extreme values because of the small sample sizes by borrowing information from neighbouring regions.

### 2.2. Model specification

The model considered in this work for disease mapping is formulated as follows:

$$y_{i}|\lambda_{i}∼\text{Poisson}(E_{i}\lambda_{i}),\;i=1,\ldots ,n$$

and

$$\log(\lambda_{i})=\eta_{i}=m_{0}+\sum_{\;f=1}^{F}\beta_{f}X_{if}+\sum_{r=1}^{R}\rho^{r}(Z_{ir})+b_{i},$$

where *η*_*i*_ is the additive linear predictor, *m*_0_ is the intercept and *β*_*f*_ is the fixed effect of the covariate *X*_*i f*_. Random effects such as splines for nonlinear effects of covariates ***Z***_***i***_ are included through the functions ${\left\{\rho^{r}\right\}}_{r=1}^{R}$. These nonlinear effects are Gaussian models with specific covariance structures such as random walk models, autoregressive models, frailty models, spatial models and so on (see [29] for more details). The spatial effects are denoted by ***b***.

For the spatial effects, ***b***, different spatial models for areal data can be assumed, such as Besag model [30], Besag–York–Mollie (BYM) model [30], Leroux model [31] or Dean’s model [32].

The BYM model combines an unstructured random effect *v*, with precision parameter *τ*_*v*_, to capture over- or underdispersion and measurement error, often assumed as an IID term (independent and identically distributed), with a spatially structured effect *u*, with precision parameter *τ*, often assumed as a Besag term. The dependence structure of u is defined through the precision matrix *Q* as

2.7 $$Q_{ii}=\tau n_{i}\;\text{and}\;Q_{ij,i∼j}=-\tau ,$$

where *i* ∼ *j* denotes the neighbourhood of region *i*, *n*_*i*_ denotes the number of neighbours of region *i* and *τ* is a precision parameter. A traditional Besag model is improper by construction since it is intrinsic, so a proper version of the Besag model has been proposed such that the precision matrix is full rank by adding a small value to the diagonal, as follows:

2.8 $$Q_{ii}=\tau (n_{i}+d)\;\text{and}\;Q_{ij,i∼j}=-\tau ,$$

with *d* > 0 for an non-intrinsic model and *d* = 0 for the intrinsic version.

Each term in the BYM model has a precision parameter but these cannot be compared directly since they are only precision parameters and not the marginal precisions. For interpretability and identifiability, a reparametrization of the BYM model was proposed by Simpson *et al.* [33], as follows:

2.9 $$b_{i}=\frac{1}{\sqrt{\tau_{b}}}\left(\sqrt{\phi }u_{i}^{*}+\sqrt{1-\phi }v_{i}^{*}\right),$$

where *ϕ* is a weight parameter interpreted as the proportion variation explained by the spatially structured term in *b*_*i*_, and u* and v* are scaled to have a precision matrix with a generalized variance of 1 as proposed by Sørbye & Rue [34].

In the framework of this disease mapping model, we define the latent field as

$$\Omega =\{m_{0},\beta ,\rho ,b\},$$

and the set of hyperparameters ***θ*** are cascaded from the fixed effect precisions *τ*_*m*_, $\tau_{\beta }$ and parameters from the random effects ***ρ*** as well as those from ***b***.

From this construction, the data ***y*** are conditionally independent given the latent field and the hyperparameters, such that the likelihood function is

2.10 $$\pi (\Omega ,\theta |y)=\prod_{i=1}^{n}f\;(y_{i}|\Omega ,\theta ),$$

such that the linear predictors are linked to the latent field through the design matrix ***A*** as

η=AΩ.

### 2.3. Prior specification and posterior propriety

We assume prior independence among the parameters and, as such, we assign centred Gaussian priors with specific precisions to the latent field elements, and various other priors to the hyperparameters as set out next.

For the latent field elements, we assume the following:

2.11 $$\begin{array}{rl} & m_{0}∼N(0,\tau_{m}),\;\;\;\beta |\tau_{\beta }∼N(0,\tau_{\beta }I)\\ \;\mathrm{and}\; & \rho |\theta_{\rho }∼N(0,Q_{\rho }),\;b|\theta_{b}∼N(0,Q_{b}), \end{array}$$

so that the joint prior for this part of the latent field is

Ω|θ∼N(0,Q(θ)),

where $\Omega ={\left(m_{0},\beta ,\rho ,b\right)}^{T}$ is the latent field, $\theta =\{\tau_{m},\tau_{\beta },\theta_{\rho },\theta_{b}\}$, and the precision matrix ***Q***(***θ***) has a block diagonal structure as formed from (2.11). The structure of $Q_{\rho }$ is determined by the specific random effects like an autoregressive model or a spline model while ***Q***_*b*_ is constructed to reflect the BYM or proper Besag model structure as in (2.9) and (2.8), respectively.

The vector of hyperparameters, θ, is assigned a joint prior π(θ), which is composed of independent marginal proper priors of any shape (not necessarily Gaussian). Often we use penalizing complexity priors to ensure against overfitting [33].

The joint posterior of the unknown parameters Ω and θ from (2.10) and (2.11) is

π(Ω,θ|y)∝π(y|Ω,θ)π(Ω|θ)π(θ),

and based on the prior structures, the posterior propriety holds.

### 2.4. Approximate inference using INLA

Computational Bayesian inference can be achieved largely in one of two ways, either through sampling-based methods like MCMC and deviants or approximately using approximate methods like variational methods or Laplace approximations like the INLA. INLA, as introduced by Rue *et al.* [24], has been shown to be widely applicable to various statistical models; in particular, to the latent Gaussian models class in which disease mapping models are included [35–38].

INLA employs a series of Laplace approximations and numerical integration to perform approximate Bayesian inference through numerically approximating the posterior densities of the latent field and hyperparameters. Since its inception, various advances have been proposed and the latest techniques in the INLA methodology are described by Van Niekerk *et al.* [25] and Gaedke-Merzhäuser *et al.* [26]. For convenience, we briefly summarize the methodology.

For data ***y***, latent field Ω, and hyperparameters ***θ***, the INLA methodology can be summarized as follows:

(i) Find the *m*-variate Gaussian approximation of π(Ω|θ,y) at the mode μ(θ) of π(Ω|θ,y), with matching curvature using the Hessian of π(Ω|θ,y) at the mode μ(θ).
(ii) Let
  2.12 $$\tilde{\pi }(\theta |y)\propto {\frac{\pi (\Omega^{*},\theta |y)}{\pi_{G}(\Omega^{*}|\theta ,y)}|}_{\Omega^{*}=\mu (\theta )}.$$
  We locate the mode of $\tilde{\pi }(\theta |y)$, from which we construct a set of *T* integration points $\theta^{*}$ in the area of the highest probability mass of $\tilde{\pi }(\theta |y)$.
(iii) Calculate
  2.13 $$\tilde{\pi }(\theta_{j}|y)=\int_{\theta_{-j}}\tilde{\pi }(\theta |y)\;d\theta_{-j},$$
  where we note that this is a low-dimensional integral since *p* is generally small.
(iv) Now define
  2.14 $$\tilde{\pi }(\Omega_{i}|\theta_{k}^{*},y)\approx {\frac{\pi (\Omega^{*},\theta_{k}^{*}|y)}{\pi_{G}(\Omega_{-i}^{*}|\Omega_{i},\theta_{k}^{*},y)}|}_{\Omega_{-i}^{*}=\mu_{-i}(\theta_{k}^{*})},$$
  with $\pi_{G}(\Omega_{-i}^{*}|\Omega_{i},\theta_{k}^{*},y)$ the (*m* − 1)-variate Gaussian approximation at the mode $\mu_{-i}(\theta )$ for the *T* configuration points $\theta_{k}^{*},k=1,2,\ldots ,T$, and calculate the posterior marginal densities of the latent field as
  2.15 $$\tilde{\pi }(\Omega_{i}|y)\approx \sum_{k=1}^{T}\tilde{\pi }(\Omega_{i}|\theta_{k}^{*},y)\tilde{\pi }(\theta_{k}^{*}|y)\Delta_{k},$$
  where $\tilde{\pi }(\theta_{k}^{*}|y)$ is from step (iii), with Δ_*k*_ the step size.

Various simplifications to the approximations have been proposed as well in order to achieve increased computational efficiency, such as an empirical Bayes approach where the integration points $\theta_{k}$ are all set to the mode of $\tilde{\pi }(\theta |y)$, termed the *simplified Laplace approximation strategy*. A recent advance is to use only the first Laplace approximation in step (ii). Then, instead of the second Laplace approximation in step (iv), the univariate conditional posteriors of $\Omega_{i}$ are crudely extracted from aforementioned Laplace approximation, whereafter a mean and variance variational Bayes correction is employed to improve the accuracy. These details can be found in [25].

### 2.5. Joint disease mapping

Sometimes specific diseases have similar spatial patterns due to sharing the same aetiologies. In this case, these diseases have some dependence, and it would be naive and misleading to model them separately. Thus, joint disease mapping is proposed to obtain improved estimates and model dependence in an appropriate manner. There are two common joint disease mapping approaches. Firstly, ecological regression, where the rate of one disease enters as a covariate in the risk regression of another disease. This approach assumes that the risk is measured without any error. Secondly, the shared component model (SCM), where a shared component is included in the risk regression of both diseases and information of both diseases is used to estimate the models. The joint disease mapping model based on a SCM for two diseases (the extension is trivial for more diseases) can be formulated for region *i* as

$$\begin{array}{rl} & y_{i1}|\lambda_{i1}∼\text{Poisson}\;(E_{i1}\lambda_{i1}),\\ & y_{i2}|\lambda_{i2}∼\text{Poisson}\;(E_{i2}\lambda_{i2}),\\ & \log(\lambda_{i1})=m_{1}+\sum_{\;f=1}^{F_{1}}\beta_{f}X_{if}+\sum_{r=1}^{R_{1}}\rho^{r}(u_{ir})+b_{i1}+S_{i}\\ \;\mathrm{and}\; & \log(\lambda_{i2})=m_{2}+\sum_{\;f=1}^{F_{2}}\gamma_{f}Z_{if}+\sum_{r=1}^{R_{2}}\xi^{r}(v_{ir})+b_{i2}+aS_{i}, \end{array}$$

where *m*_*d*_ is a disease-specific intercept for disease *d*, *β*_*f*_ is the *f*th fixed effect for disease 1 with covariate $X_{f}$, *γ*_*f*_ is the *f*th fixed effect for disease 2 with covariate $Z_{f}$, *ρ*^*r*^ is the *r*th random effect of disease 1 with covariate $u_{r}$, *ξ*^*r*^ is the *r*th random effect of disease 2 with covariate $v_{r}$, *b*_*i d*_ is a disease-specific spatial random effect (a BYM or Besag term), and *S*_*i*_ is the shared spatial random effect with a proper Besag prior as in (2.8) with parameters *τ* and *d*. Here, *a* and S can be used to evaluate the spatial dependence between the two diseases while ***b***_*d*_ presents the spatial dependence within each disease. The prior we assume for *a* is Gaussian with mean 0 and variance 1. All other priors are assigned as described in §2.3.

In the framework of joint disease mapping we have a larger latent field since we define Ω as

$$\Omega =\{m_{1},m_{2},\beta ,\gamma ,\rho ,\xi ,b_{1},b_{2},S\},$$

and again we collect all hyperparameters in θ, including *a*. By defining this extended latent field and hyperparameter set, the model can be identified as another latent Gaussian model; thus, we can use INLA for the Bayesian inference thereof. We omit the technical details of the latent Gaussian model development for joint models (models with more than one likelihood and regression model) and refer the interested reader to Van Niekerk *et al.* [39] for these details.

### 2.6. Simulation study

The code for this simulation study is available at https://github.com/JanetVN1201/Code_for_papers/tree/main/Joint%20quantile%20disease%20mapping%20.

In this simulation study, we simulate two sets of data: one in which the two diseases are correlated and another in which the diseases are independent. We use the regions of Pennsylvania as our area of interest; there are 67 connected regions within this area. The model that we postulate is

2.16 $$\begin{array}{rl} & y_{i1}|\lambda_{i1}∼\text{Poisson}\;(\lambda_{i1})\;\text{with}\;\log(\lambda_{i1})=m_{1}+b_{i1}+S_{i}\\ \;\mathrm{and}\; & y_{i2}|\lambda_{i2}∼\text{Poisson}\;(\lambda_{i2})\;\text{with}\;\log(\lambda_{i2})=m_{2}+b_{i2}+aS_{i}, \end{array}$$

where $b_{1}$, $b_{2}$ and S are proper Besag terms as in (2.8) with parameters *τ*_1_, *d*_1_, *τ*_2_, *d*_2_, *τ* and *d*, respectively. The assigned vague priors are

$$m_{1},m_{2}∼N(0,1000),\;\tau_{1},\tau_{2},\tau ∼\text{Gamma}(1,0.0005)$$

and

$$d_{1},d_{2},d∼\text{Gamma}(1,1),\;a∼N(0,1000).$$

The correlated data are simulated based on the following relative risk models:

$$y_{i1}|\lambda_{i1}∼\text{Poisson}\;(\lambda_{i1})\;\text{with}\;\log(\lambda_{i1})=1+S_{i}$$

and

$$y_{i2}|\lambda_{i2}∼\text{Poisson}\;(\lambda_{i2})\;\text{with}\;\log(\lambda_{i2})=1+0.7S_{i},$$

where *S*_*i*_ is the shared component that follows a proper Besag model (2.8) with precision parameter *τ* = 1 and *d* = 1.

**Figure 1.**
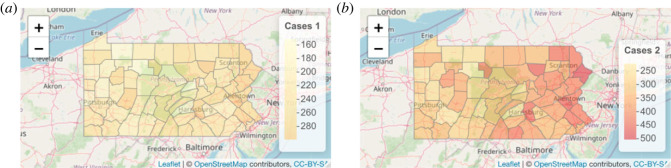
One realization of cases of disease 1 (*a*) and disease 2 (*b*) under the correlated setup by using joint disease mapping.

Figure 1 shows one realization of the simulated correlated data. We considered 20 realizations as our dataset (without loss of generality). Even though the data are simulated without disease-specific spatial effects, we include the disease-specific spatial effects in our model to ascertain if the model can distinguish between the two sources of spatial variation (shared and non-shared).

The independent data were generated as follows:

$$\begin{array}{cc} y_{i1}|\lambda_{i1}∼\text{Poisson}\;(\lambda_{i1}) & \text{with}\;\log(\lambda_{i1})=1+b_{i1}\\ y_{i2}|\lambda_{i2}∼\text{Poisson}\;(\lambda_{i2}) & \text{with}\;\log(\lambda_{i2})=1+b_{i2}, \end{array}$$

where *b*_*i*1_ and *b*_*i*2_ are proper Besag terms with parameters *τ*_1_ = 1, *d*_1_ = 1, *τ*_2_ = 1 and *d*_2_ = 1.

The estimated values of the parameters obtained by INLA are similar to the true values, as shown in table 1. Note that the estimated values of *τ*_1_ and *τ*_2_ are very large for the correlated data, indicating a very small marginal variance of the disease-specific spatial effect and thereby negating the inclusion of these effects as expected. For the independent data, the estimated value of *τ* is large hence negating the inclusion of the shared component.

**Table 1.** Posterior inference for model (2.16) with correlated and independent data.

	correlated data			independent data		
parameter	true	mean	95% credible interval	true	mean	95% credible interval
*m*_1_	1	1.11	(1.020; 1.776)	1	1.109	(1.015; 1.202)
*m*_2_	1	1.029	(0.958; 1.099)	1	0.965	(0.865; 1.065)
*τ*_1_	—	2060	(257.9; 8120)	1	1.33	(0.869; 1.96)
*d*_1_	—	1.165	(0.139; 3.92)	1	0.823	(0.299; 1.84)
*τ*_2_	—	2674	(338.4; 1100)	1	1.423	(0.914; 2.10)
*d*_2_	—	1.515	(0.088; 7.17)	1	0.682	(0.237; 1.60)
*τ*	1	1.236	(0.778; 1.81)	—	2955	(3.641; 20763)
*d*	1	1.089	(0.384; 2.68)	—	1.195	(0.235; 3.15)
*a*	0.7	0.703	(0.553; 0.849)	—	1.006	(0.432; 1.62)

The deviance information criterion (DIC) and the Watanabe–Akaike information criterion (WAIC) [40] are presented in table 2. These model selection criteria show a preference for the joint model when the data are correlated and a preference for separate models, which is (2.16) without the shared components S, when the data are independent. This indicates stable estimation and the model’s ability to distinctly estimate an associated joint model if needed, while not overfitting when the model should not have been specified jointly.

**Table 2.** Model selection criteria for model (2.16) with correlated and independent data; italics indicate the better fit.

	correlated data		independent data	
model	DIC	WAIC	DIC	WAIC
model for *λ*_*i*1_ only	5515	5502	5498	5498
model for *λ*_*i*2_ only	5199	5208	5457	5444
sum of theseparate models	10714	10710	*10955*	*10942*
joint mean model	*10543*	*10519*	10985	10964

## 3. Model-based quantile regression

### 3.1. Introduction

Quantile regression models the conditional quantile of the response variable given the explanatory variables, instead of the conditional mean. Let *Y* be a real valued random variable. The *α*th quantile of *Y* is given by

$$Q(\alpha )=F^{-1}(\alpha )=\text{inf}\{y\;:\;F(y)\geq \alpha \}\;\text{for}\;0\leq \alpha \leq 1,$$

where *F*(*y*) = *P*(*Y* ≤ *y*) is the cumulative distribution function (CDF) of the random variable *Y*. As in mean regression, a loss function is used in order to infer the parameters. The loss function most often used in the framework of quantile regression is the check loss function. Given that 0 ≤ *α* ≤ 1, ∀u∈R, the quantile loss function is defined as

$$\rho_{\alpha }(u)=\left\{ \begin{array}{cc} u\alpha & u\geq 0\\ u(\alpha -1) & u<0. \end{array}\right.$$

An estimate of the *α*th quantile of the random variable *Y* can be obtained by minimizing the following risk function:

3.1 $$\underset{q_{\alpha }\in R}{\text{argmin}}\;E\;[\rho_{\alpha }(Y-q_{\alpha })],$$

where *q*_*α*_ is the *α*th quantile of the random variable *Y*. However, when the quantile $q_{\alpha }$ is influenced by the explanatory variables **X** ∈ *R*^*p*^, it is referred to as the *α*th conditional quantile. This relationship can be articulated as $q_{\alpha }=Q_{\alpha }(Y|X)$, which can be formally represented by

$$Q_{\alpha }(Y|X)=X^{⊤}\beta_{\alpha },$$

where the vector $\beta_{\alpha }$ encompasses the coefficients that need estimation for the *α*th quantile. The estimate of the conditional quantile is called quantile regression. The quantile regression models the relationship between **X** and the quantile of *Y*. The estimate of the quantile regression can be written as

3.2 $${\hat{q}}_{\alpha }=\underset{q_{\alpha }\in R}{\text{argmin}}\;E\;[\rho_{\alpha }(Y-q_{\alpha })],$$

such that $F({\hat{q}}_{\alpha })=\alpha$. This approach encompasses the use of the ALD for quantile regression in a non-parametric fashion since the optimization in (3.2) is independent of the shape of *F*. The use of the ALD likelihood is justified by the fact that the corresponding maximum-likelihood estimator coincides with the optimum defined in (3.1). However, although this choice may seem appealing due to the apparently weak modelling assumption on the response, the ALD may not represent the shape of the data; thus it is a working likelihood and not a generative one. Adopting the ALD imposes several restrictions that may not be obvious or desirable in applications: the skewness of the density is fully determined when a specific percentile is chosen, the density is symmetric when *α* = 0.5, and the mode of the error distribution is at 0 regardless of *α*, which results in rigid error density tails for extreme percentiles [41].

The limitations of using a working likelihood are even more critical in the Bayesian framework, where the lack of a generating likelihood implies that the validity of posterior inference is no longer guaranteed by the Bayes theorem. As shown by Yang *et al.* [42], the scale parameter of the ALD affects the posterior variance, despite not having any impact on the quantile itself, meaning that posterior credibility intervals are not stable. Although some corrections for the posterior variance can be found in the literature, for example in [42], these results are only asymptotically valid.

In the case of disease mapping, we *do not* have a real valued response variable *Y*, resulting in a discontinuous quantile function *Q*(*α*). Thus the optimization in (3.2) leads to multiple solutions. Various proposals to induce smoothness in the quantile function can be found in the literature by mainly either jittering or interpolation. However, these approaches often lead to quantile crossing since a new smoothing is needed for each *α*. Liu *et al.* [22] proposed a discrete version of the ALD for discrete data, which also induces quantile crossing unless multiple quantiles are modelled jointly, which is not feasible with their MCMC based approach.

Often, the response variable distribution is specified parameterically and is not in question, like in disease mapping. In geostatistics, Leiva *et al.* [43] propose a quantile regression model based on the Birnbaum–Saunders distribution often used in this field. When the parametric assumptions are not challenged, a model-based approach to quantile regression seems more intuitive than invoking an approximate likelihood with extra unknown parameters. Moreover, by linking the quantile to the canonical parameter of the distribution, we can gain more insight into the data itself.

We focus on the case where we can assume the true generating model is known. As opposed to mean regression, where generalization of the basic linear model heavily relies on the response distribution, in quantile regression, this is a relatively unexplored strategy, with the notable exceptions of Noufaily & Jones [44], Opitz *et al.* [45], Castro-Camilo *et al.* [46] and Frumento & Salvati [47]. We refer to this setting as *model-based quantile regression*.

For a principled Bayesian analysis using quantile regression for discrete data, we need an approach that respects the form of the underlying data, is not prone to quantile crossing, and is computationally efficient to implement. Hence, we propose model-based quantile regression.

### 3.2. Proposal

Model-based quantile regression is an approach for quantile regression that considers the quantiles of the generating distribution instead of a working likelihood. This approach extends the generalized linear mixed model (GLMM) framework from modelling means to modelling quantiles. Our proposal is comprised of two stages: firstly, a generalized additive mixed model (GAMM) for the quantile with an invertible link function *g*(), and secondly, the quantile is mapped to the canonical parameter of the distribution through a mapping function *h*().

This approach can be used in both frequentist and Bayesian frameworks. The parameters of a model-based quantile regression model are all identifiable, which is not the case when the ALD and its variations are employed.

Let *F*(*y*_*i*_, *λ*_*i*_) be the distribution of *Y*_*i*_|*X*_*i*_, for *i* = 1, …, *n*, where *λ*_*i*_ is the canonical parameter of the distribution. Given 0 ≤ *α* ≤ 1, the *α*th quantile of *Y*_*i*_|*X*_*i*_ is *q*_*i*,*α*_ = *Q*_*α*_ (*Y*_*i*_|*X*_*i*_). The model-based quantile regression model is constructed as follows:

**Step 1. *Modelling***

The quantile *q*_*i*,*α*_ of the distribution *F*(*y*_*i*_, *λ*_*i*_) is modelled as follows:

$$q_{i,\alpha }=g(\eta_{i,\alpha }),$$

where *g* is an invertible function, and *η*_*i*,*α*_ is the linear predictor for the *α*th quantile of the response variable for the *i*th observation. The linear predictor can include fixed effects, random effects or both. Moreover, parametric or semi-parametric models can be included in this approach in order to study the impact of the covariates at different levels of the distribution and non-parametric models can be used for prediction.

**Step 2. *Mapping***

The quantile *q*_*i*,*α*_ is mapped to the parameter *λ*_*i*_ of the distribution *F*(*y*_*i*_, *λ*_*i*_) as

3.3 $$\lambda_{i,\alpha }=h(q_{i,\alpha }),$$

where *h* is an invertible mapping function. This function *h* is derived by inverting the CDF of *F*(*y*_*i*_, *λ*_*i*_) to obtain the quantile function and subsequently expressing *λ*_*i*_ as a function of the quantile.

In this approach, the parameter *λ*_*i*_ is modelled implicitly, by explicitly modelling the quantile and invoking the functions *g*() and *h*(). Unlike mean regression, when the parameter of the generating distribution links to the linear predictor through a function *λ*_*i*_ = *g*(*η*_*i*_), where *η*_*i*_ represents the linear predictor for the expected value (mean) of the response variable for the *i*th observation, in model-based quantile regression the parameter of the generating distribution is linked to the linear predictor through a composition function *λ*_*i*,*α*_ = *h*(*g*(*η*_*i*,*α*_)).

### 3.3. Model-based quantile regression for discrete data

The extension of model-based quantile regression for discrete random variables is not straightforward, since the objective function in (3.1) is non-differentiable for discrete random variables. The positive mass of the points for the discrete variable prevents the sample quantile from having an asymptotic distribution. Additionally, it is not easy to apply the modelling and mapping steps of model-based quantile regression, as described in §3.2, to discrete data. First, in the modelling step, the common models for *g* are the log for count data and the logit for binary data; these are continuous functions. Therefore, the model *q*_*i*,*α*_ = *g*(*η*_*i*,*α*_) is not appropriate, since the quantile on the left-hand side is discrete whereas the function *g* is continuous. Additionally, the map *h* is troublesome to derive since the CDF is non-invertible, which implies that there is no unique *λ*_*i*_ to generate each quantile.

To address these issues, we approximate the distributions for discrete data by deriving continuous counterparts, and then model the quantiles of the continuous version instead of the discrete. The continuous counterpart is obtained by interpolating the CDF of the discrete random variable in a way that respects the original shape. The model-based quantile method can be applied to discrete variables if their CDF can be expressed as

$$F_{Y}(y,\lambda )=P(Y\leq y)=k(\lfloor y\rfloor ,\lambda ),$$

where *k* is a continuous function, and *Y* is a discrete random variable. The interpolation can be obtained by removing the floor operator, so that *k*(*y*, *λ*) is the CDF of the continuous version of *Y*, denoted by *Y*^′^. By the definition of the floor operator, for all integers *y*,

3.4 $$F_{Y}(y,\lambda )=k(\lfloor y\rfloor ,\lambda )=k(y,\lambda )=F_{Y^{\prime }}(y,\lambda ).$$

The (continuous) distribution of *Y*′ is considered as a continuous generalization of the discrete variable *Y* because the respective CDFs are equal for all integer values *y*. While modelling the quantiles of a continuous approximation implies that the fitted quantiles curves are not discrete, the equivariance property of quantile guarantees that

3.5 $$Q_{\alpha }(Y|X)=Q_{\alpha }(\lceil Y^{\prime }\rceil |X)=\lceil Q_{\alpha }(Y^{\prime }|X)\rceil ,$$

where **X** are explanatory variables ∈*R*^*p*^. We highlight that our proposal allows for any type of inferential procedure on the quantile curves, as opposed to other likelihood-based methods working on the ALD assumption, which are limited to point estimation. Hypothesis testing and confidence intervals can be approached asymptotically by deriving a limiting distribution for the estimator of the quantile curves. In order to do so, it is enough to note that the link function obtained by composition of the modelling and mapping steps is monotone and differentiable; as a consequence, it follows directly from the delta method that the maximum-likelihood estimators of the quantile curves ${\hat{Q_{\alpha }}}^{\left(\mathrm{MLE}\right)}(Y|X)$ for every *α* are asymptotically Gaussian [48].

In the next section, we present the details for count data assumed to follow a Poisson distribution. The details for binomial and negative binomial data are provided in appendix A.

#### 3.3.1. Continuous Poisson

Here, we present details on the approximation of the discrete Poisson distribution with a continuous Poisson (CP) counterpart.

The CDF of a Poisson distribution can be expressed as the ratio of an incomplete and regular Gamma function, as follows:

3.6 $$Y|\lambda ∼\text{Poisson}(\lambda )\;F_{Y}(y)=P(Y\leq y)=\frac{\Gamma (\lfloor y\rfloor +1,\lambda )}{\Gamma (\lfloor y\rfloor +1)}\;y\geq 0$$

where $\Gamma (y,\lambda )=\int_{\lambda }^{\infty }e^{-s}s^{y-1}\;ds$ is the upper incomplete Gamma function. Following §3.3, the CP is then defined from (3.6) as

$$Y^{\prime }|\lambda ∼\text{Continuous Poisson}(\lambda )\;F_{Y^{\prime }}(y)=P(Y^{\prime }\leq y)=\frac{\Gamma (y+1,\lambda )}{\Gamma (y+1)}\;y>-1.$$

**Figure 2.**
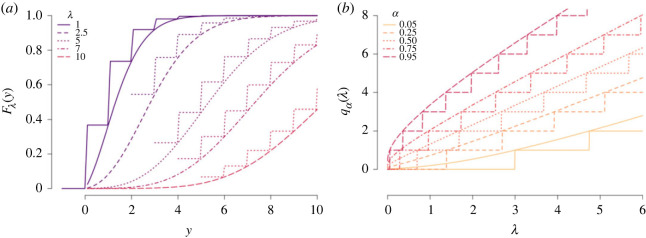
CDF (*a*) and quantile functions (*b*) of the discrete and continuous Poisson.

The reason for changing the support from *y* ≥ 0 to *y* > −1 is to avoid mass at 0, so there will be no jump in the CDF of the CP, as illustrated in figure 2. The CP and discrete Poisson random variables can be related as $Y=\lceil Y^{\prime }\rceil$. The CDF and quantile function of the Poisson and CP are illustrated in figure 2 for specific values of *y*, *λ* and *α*, and the properties in (3.4) and (3.5) are evident. The model-based quantile regression model for Poisson data is then defined for *Y*_*i*_|*η*_*i*_, a continuous Poisson random variable with parameter *λ*_*i*_, as

3.7 $$\begin{array}{rl} & q_{i,\alpha }=g(\eta_{i,\alpha })=\exp\{\eta_{i,\alpha }\}\\ \;\mathrm{and}\; & \lambda_{i,\alpha }=h(q_{i,\alpha })=\frac{\Gamma^{-1}(q_{i,\alpha }+1,1-\alpha )}{\Gamma (q_{i,\alpha }+1)}. \end{array}$$

#### 3.3.2. Model-based quantile regression for disease mapping

From §§3.2 and 3.3, we can define a model-based quantile regression model for disease mapping. One issue that remains is how to decompose the expected number of cases into the local expectation, *E*_*i*_, and the relative risk *λ*_*i*_. In the case of modelling the quantile instead of the mean, there are two options:

— Include *E*_*i*_ in the linear model as an offset:
  3.8 $$\begin{array}{rl} & q_{i,\alpha }=\exp\{\eta_{i,\alpha }+\log(E_{i})\}=E_{i}\exp\{\eta_{i,\alpha }\}\\ \;\mathrm{and}\; & \lambda_{i,\alpha }=\frac{\Gamma^{-1}(q_{i,\alpha }+1,1-\alpha )}{\Gamma (q_{i,\alpha }+1)}. \end{array}$$
— Consider *E*_*i*_ as a scaling of the parameter of the distribution:
  3.9 $$\begin{array}{rl} & q_{i,\alpha }=\exp\{\eta_{i,\alpha }\}\\ \;\mathrm{and}\; & \lambda_{i,\alpha }=E_{i}\frac{\Gamma^{-1}(q_{i,\alpha }+1,1-\alpha )}{\Gamma (q_{i,\alpha }+1)}. \end{array}$$

These two approaches are equivalent in Poisson mean regression, but not equal in Poisson quantile regression; thus, the choice of approach depends on the purpose of the analysis. If the focus of the study is to infer a quantile-specific model, then (3.8) is more appropriate; whereas (3.9) can be considered as a model for the parameter *λ*_*i*_.

### 3.4. Properties

As mentioned, various approaches exist for the modelling of quantiles of discrete data. Here, we pose our proposal of model-based quantile regression against the approach of Machado & Silva [20] which is based on jittering as a means of interpolation embedded in the R package *Qtools*. The code for this investigation is available at https://github.com/JanetVN1201/Code_for_papers/tree/main/Joint%20quantile%20disease%20mapping%20.

**Figure 3.**
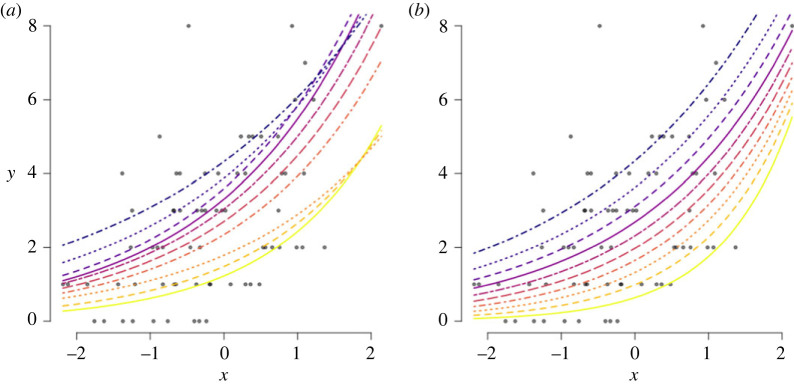
The jittering (*a*) and model-based (*b*) quantile regression fitted curves of simulated data (dots) for various different quantile levels

As a toy example, we simulate a sample of size 70 from a Poisson distribution with

$$\lambda_{i}=\exp(1+0.5X_{i}),$$

where *X*_*i*_ was simulated from a standard Gaussian distribution. We fit the following model:

$$q_{i,\alpha }=\exp(m+\beta X_{i}),$$

for various 0 < *α* < 1, using both approaches and the results are displayed in figure 3. The quantile crossing that results from the jittering approach is clear, while this does not occur with the model-based approach. Since the model-based quantile regression uses the data-generating distribution as information, each quantile regression is informed about the other quantiles, without explicitly modelling all quantile levels jointly. To overcome the quantile crossing phenomena in another framework, like jittering or the discrete ALD, multiple quantile regressions should be fitted simultaneously and a spline model can be used to smooth over them, as suggested by Wei *et al.* [49]. This is a cumbersome and time-inefficient process for a practitioner, that can be circumvented by adopting the model-based quantile regression model.

**Figure 4.**
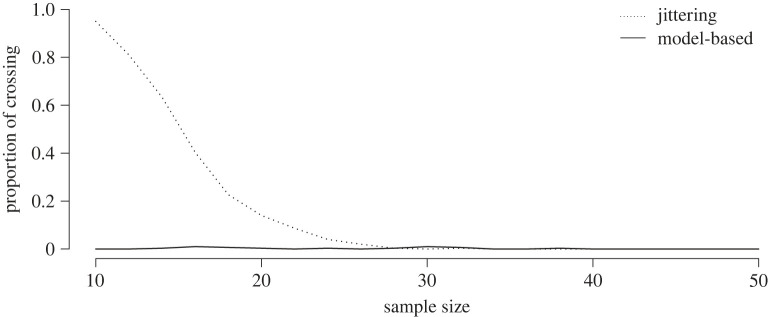
Proportion of quantile crossings based on 300 datasets for both approaches.

As a further analysis, we simulated 300 datasets, each with sample size 70 from a Poisson distribution with

$$\lambda_{i}=\exp(X_{i}),$$

where *X*_*i*_ is the absolute value of a centred Gaussian random variate with standard deviation 1.5. For each dataset, different levels (*α* ∈ {0.05, 0.1, …, 0.9, 0.95}) of the jittering and model-based quantile regression models are fitted of the form

$$q_{i,\alpha }=\exp(\beta X_{i}),$$

and the number of quantile crossings are observed. This result is displayed in figure 4, and again the robustness against quantile crossing of the model-based quantile regression approach is clear, even for small sample sizes (which is often the case in disease mapping).

## 4. Bayesian joint quantile disease mapping

The main goal of disease mapping is to estimate the relative risk of diseases across regions. Specific diseases sometimes have similar spatial patterns due to sharing the same aetiologies. In this case, these diseases have some dependence; thus, it would be more appropriate to model them jointly rather than separately. Moreover, sometimes the dependence might be in different quantiles between the diseases or some diseases could inhibit the occurrence of other diseases. The proposed joint quantile disease mapping model links different quantiles of multiple diseases using a more general framework by considering dependence not on the mean, but on the quantiles.

### 4.1. Model specification

We present the details for two diseases, although our proposal holds for analysis of more than two diseases. The joint quantile model for two diseases can be formulated as follows:

4.1 $$\begin{array}{rl} & y_{i1}|\lambda_{i1}∼\text{Poisson}\;(E_{i1}\lambda_{i1})\\ & y_{i2}|\lambda_{i2}∼\text{Poisson}\;(E_{i2}\lambda_{i2})\\ & \log(q_{i1,\alpha_{1}})=\eta_{i1,\alpha_{1}}=m_{1}+\sum_{\;f=1}^{F_{1}}\beta_{f}X_{if}+\sum_{r=1}^{R_{1}}\rho^{r}(u_{ir})+b_{i1}+S_{i}\\ \;\mathrm{and}\; & \log(q_{i2,\alpha_{2}})=\eta_{i2,\alpha_{2}}=m_{2}+\sum_{\;f=1}^{F_{2}}\gamma_{f}Z_{if}+\sum_{r=1}^{R_{2}}\xi^{r}(v_{ir})+b_{i2}+c\;S_{i}, \end{array}$$

where *λ*_*i k*_ is the relative risk of unit *i* for disease *k* and is mapped to the *α*_*k*_ level quantile $q_{ik,\alpha_{k}}$, as in §3.3.2. In the modelling part, *m*_*k*_ is a disease-specific intercept, *b*_*i k*_ is a disease-specific spatial random effect, and *S*_*i*_ is the shared spatial component following a proper Besag prior as in (2.8) with parameters *τ* and *d* with *c* as the parameter that accounts for the correlation between the two diseases. The model also incorporates fixed effects of covariates $X_{i}$ and $Z_{i}$, respectively, using β and γ, for the two diseases. Various random effects such as splines for nonlinear effects of covariates $u_{i}$ and $v_{i}$ are included through functions ${\left\{\rho^{r}\right\}}_{r=1}^{R_{1}}$ and ${\left\{\xi^{r}\right\}}_{r=1}^{R_{2}}$, respectively.

Since a composite link function is now used to map the data to the linear predictor, as in §3.2, instead of a simple link function as in joint disease mapping through mean regressions as in §2.5, many results from §2 hold in terms of model specification as in §2.2, prior specifications and posterior propriety as in §2.3.

We define the latent field

$$\Omega =\{m_{1},m_{2},\beta ,\gamma ,\rho ,\xi ,b_{1},b_{2},S\},$$

and hyperparameters $\theta =\{c,\tau ,d,\theta_{\rho },\theta_{\xi },\theta_{b_{1}},\theta_{b_{2}},\ldots \}$, whereafter the data $y=\{y_{1},y_{2}\}$ are conditionally independent given the latent field and the hyperparameters, such that the likelihood function can be expressed as

4.2 $$\pi (\Omega ,\theta |y)=\prod_{i=1}^{n}f\;(y_{i}|\Omega ,\theta ).$$

As in §2.3, the prior for the latent field, π(Ω|θ), is Gaussian by construction with a block diagonal precision matrix Q(θ), and the prior for the hyperparameters, π(θ), is composed from multiple independent priors to form the joint posterior of Ω and θ from (4.2) is

π(Ω,θ|y)∝π(y|Ω,θ)π(Ω|θ)π(θ),

with linear predictors η=AΩ, and based on the proper priors, the posterior propriety holds.

We use the INLA framework to perform approximate Bayesian inference of this model (4.1), as mentioned in §2.4, to avoid the computational cost of MCMC while maintaining the accuracy of the posterior estimates.

### 4.2. Simulation study

The code for this simulation study is available at https://github.com/JanetVN1201/Code_for_papers/tree/main/Joint%20quantile%20disease%20mapping%20.

In this section, we simulate independent and correlated data based on the map of Pennsylvania, which is considered as a connected graph of size 67. The model we fit here is

4.3 $$\begin{array}{rl} & y_{i1}|\lambda_{i1}∼\text{Poisson}\;(\lambda_{i1})\;\text{with}\;\log(q_{i1,\alpha_{1}})=m_{1}+b_{i1}+S_{i}\\ \;\mathrm{and}\; & y_{i2}|\lambda_{i2}∼\text{Poisson}\;(\lambda_{i2})\;\text{with}\;\log(q_{i2,\alpha_{2}})=m_{2}+b_{i2}+cS_{i}, \end{array}$$

where $b_{1}$, $b_{2}$ and S are proper Besag terms, as in (2.8), with parameters *τ*_1_, *d*_1_, *τ*_2_, *d*_2_, *τ* and *d*, respectively. We assume the following vague priors:

$$\begin{array}{cc} m_{1},m_{2}∼N(0,1000), & \tau_{1},\tau_{2},\tau ∼\text{Gamma}(1,0.0005)\\ d_{1},d_{2},d∼\text{Gamma}(1,1), & c∼N(0,1000). \end{array}$$

The correlated data were generated based on the following quantile regression models:

$$y_{i1}|\lambda_{i1}∼\text{Poisson}\;(\lambda_{i1})\;\text{with}\;\log(q_{i1,0.2})=1+S_{i}$$

and

$$y_{i2}|\lambda_{i2}∼\text{Poisson}\;(\lambda_{i2})\;\text{with}\;\log(q_{i2,0.8})=3+0.7\;S_{i},$$

where *S*_*i*_ is the shared spatial component that follows a Besag proper model (2.8) with precision parameter *τ* = 1 and parameter *d* = 1.

**Figure 5.**
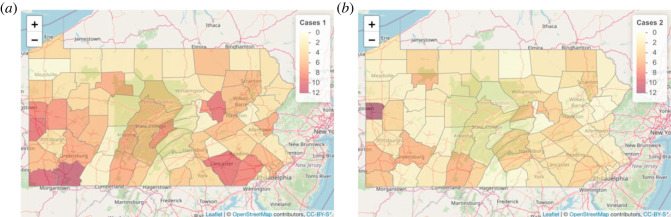
One realization of cases of disease 1 (*a*) and disease 2 (*b*) under the correlated setup by using joint quantile disease mapping.

Figure 5 shows one realization of the correlated data that were added to the Pennsylvania map.

The independent data were generated based on the following quantile regression models:

$$y_{i1}∼\text{Poisson}\;(\lambda_{i1})\;\text{with}\;\log(q_{i1,0.2})=1+b_{i1}$$

and

$$y_{i2}∼\text{Poisson}\;(\lambda_{i2})\;\text{with}\;\log(q_{i2,0.8})=3+b_{i2},$$

where *b*_*i*1_ and *b*_*i*2_ are proper Besag terms (2.8) with *τ*_1_ = 1, *τ*_2_ = 1, *d*_1_ = 1, and *d*_2_ = 1.

The estimated values of the parameters obtained by R INLA are similar to the true values, as shown in table 3. For the correlated data (where no disease-specific spatial effects were included), the large estimates of *τ*_1_ and *τ*_2_ indicate the absence of disease-specific spatial effects, since these result in very small variance. Similarly, the large value of *τ* in the case of independent data indicates a negligible shared spatial component.

**Table 3.** Posterior inference for model (4.3) with correlated and independent data.

	correlated data			independent data		
parameter	true	mean	95% credible interval	true	mean	95% credible interval
*m*_1_	1	1.118	(1.036; 1.199)	1	1.121	(1.030; 1.21)
*m*_2_	3	3.037	(2.986; 3.088)	3	2.968	(2.892; 3.043)
*τ*_1_	—	3252	(647.5; 12400)	1	1.29	(0.783; 1.98)
*d*_1_	—	0.561	(0.094; 2.15)	1	1.22	(0.379; 3.23)
*τ*_2_	—	2598	(603.4; 6690)	1	1.05	(0.849; 1.32)
*d*_2_	—	0.473	(0.167; 1.1)	1	1.05	(0.523; 1.78)
*τ*	1	0.967	(0.661; 1.33)	—	27822	(669.5; 190000)
*d*	1	1.573	(0.905; 2.68)	—	1.10	(0; 7.94)
*c*	0.7	0.735	(0.637; 0.832)	—	1.14	(0.533; 1.84)

The model selection criteria presented in table 4 show a preference for the joint quantile model when the data are correlated and a preference for the separate quantile regression models, which are (4.3) without the shared components, when the data are independent. This indicates stable estimation and the model’s ability to distinctly estimate an associated joint model, if needed.

**Table 4.** Model selection criteria for model (4.3) with correlated and independent data; italics indicate the better fit.

	correlated data		independent data	
model	DIC	WAIC	DIC	WAIC
model for *q*_*i*1,0.2_ only	3226	3239	3226	3239
model for *q*_*i*2,0.8_ only	4191	4162	4191	4161
sum of the separate models	7417	7400	*7417*	*7400*
joint quantile model	*7211*	*7198*	7615	7578

## 5. Joint quantile disease mapping model for malaria and G6PD deficiency

In this section, we fit the Bayesian joint quantile disease mapping model proposed in §4 using INLA to model the quantiles of the incidences of malaria and G6PD deficiency in some African countries as well as their dependence. The code for this analysis is available at https://github.com/JanetVN1201/Code_for_papers/tree/main/Joint%20quantile%20disease%20mapping%20.

### 5.1. Exploratory data analysis

The numbers of cases of malaria and G6PD per region were obtained from https://malariaatlas.org/. Various country-level covariates can be used in our model; however, for the motivating example, the emphasis is placed on the joint component, even though various fixed and random effects might be considered for a thorough analysis of the data itself.

**Figure 6.**
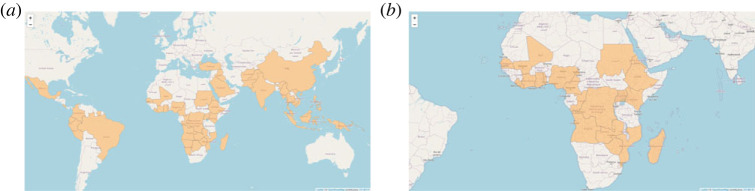
Countries where both G6PD deficiency and malaria cases are observed (*a*) on the African continent (*b*).

**Figure 7.**
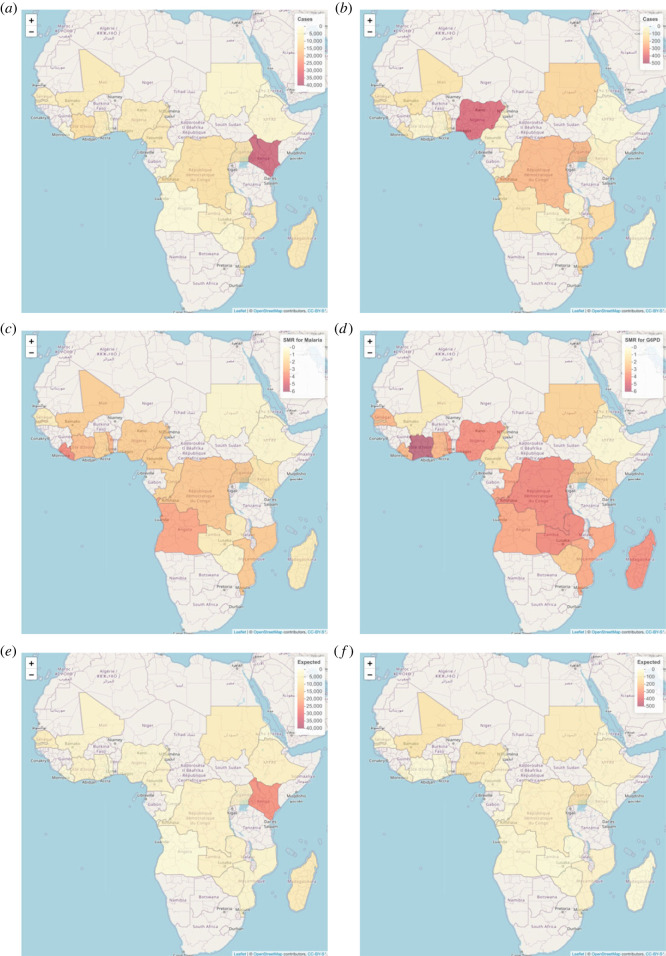
Observed number of cases (*a,b*), SMR (*c,d*) and expected number of cases (*e,f*) of malaria (*a,c,e*) and G6PD (*b,d,f*).

We only selected the countries for which information on both malaria and G6PD is available, as indicated in figure 6. According to figure 6, the countries are distributed around the world. Since we want to investigate the spatial correlation, we consider the African continent so that most countries included have some neighbours, as in figure 6. In figure 7, the SMRs for malaria and G6PD deficiency are presented. In general, the risk of G6PD deficiency is higher than the risk of malaria because G6PD deficiency has a higher SMR. Some areas such as Abidjan and Madagascar that are considered to have the highest risk of G6PD deficiency have the lowest risk of malaria according to the SMR values, which could indicate a prohibitive relationship between these two diseases. The numbers of observed cases of malaria and G6PD can be seen in figure 7. Kenya has the highest number of malaria cases, while Nigeria has the highest number of G6PD deficiency cases. We also include the expected number of cases for each country based on the assumption of similar global and local behaviour in figure 7.

### 5.2. Results

To investigate the relationship between the quantiles of malaria and G6PD deficiency, we applied the joint quantile model proposed in §4, with *y*_*i*1_ and *y*_*i*2_ representing the cases of malaria and G6PD deficiency, respectively. Thus, the proposed model is

5.1 $$\begin{array}{rl} & y_{i1}|\lambda_{i1}∼\text{Poisson}\;(E_{i1}\lambda_{i1})\\ & y_{i2}|\lambda_{i2}∼\text{Poisson}\;(E_{i2}\lambda_{i2})\\ & \log(q_{i1,\alpha_{1}})=m_{1}+b_{i1}+S_{i}\\ \;\mathrm{and}\; & \log(q_{i2,\alpha_{2}})=m_{2}+b_{i2}+c\;S_{i}, \end{array}$$

with *b*_*i*1_ and *b*_*i*2_ assumed to be distributed as a BYM model (2.9) with parameters *τ*_1_ and *ϕ*_1_, and *τ*_2_ and *ϕ*_2_, respectively, to allow for spatially structured and unstructured effects. *S*_*i*_ is assumed to be a proper Besag term as in (2.8) with parameters *τ* and *d*.

We present the results based on quantile levels *α*_1_ = 0.2 and *α*_2_ = 0.8, to model the relationship between a low quantile of malaria and a high quantile of G6PD. For illustration, we also investigated the opposite relationship, i.e. between a high quantile of malaria and a low quantile of G6PD deficiency. Other levels were also considered and the results are presented in appendix B.

**Figure 8.**
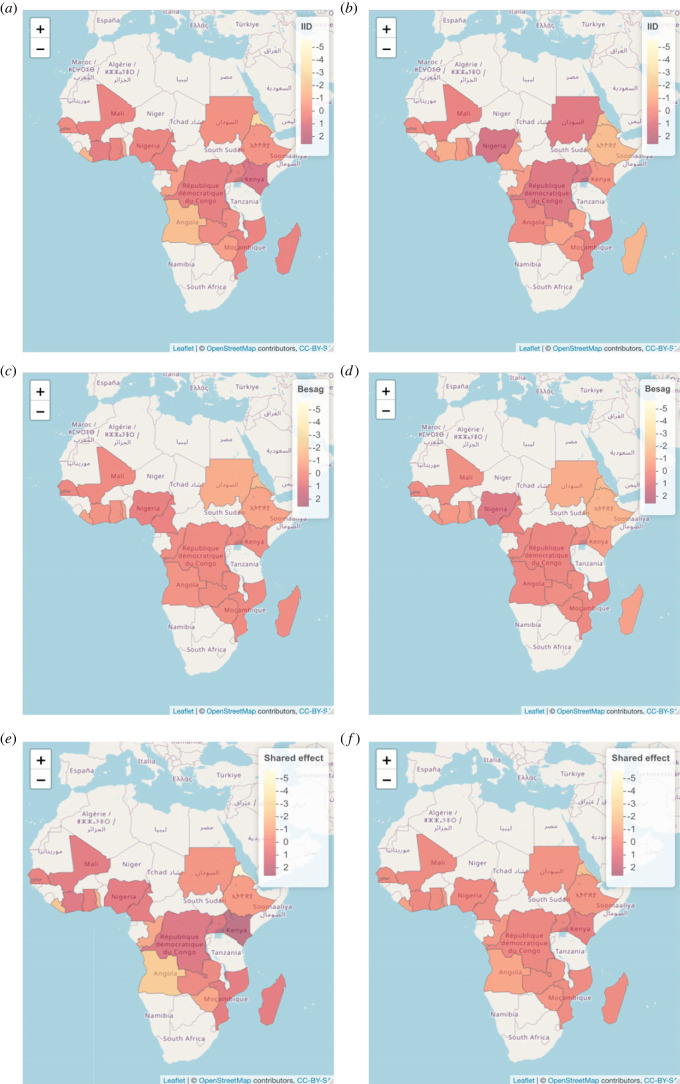
Posterior mean of the unstructured (*a,b*), structured (*c,d*), and shared (*e,f*) spatial effect in the model for malaria (*a,c,e*) and G6PD deficiency (*b,d,f*).

The posterior inference is presented in table 5. With regards to the proposed model (5.1), we note that the precision parameter for the shared spatial effect, *τ*, is small, indicating a significant spatial correlation structure. To investigate the relationship between the quantiles of the two diseases we interpret the posterior inference of *c*. Since *c* ∈ (0.012; 0.442), we can conclude that the quantiles of the two diseases with *α*_1_ = 0.2 and *α*_2_ = 0.8, share a common spatial field and are thus correlated. The positive association between the two quantiles is unexpected and indicates evidence *against* the anecdotal claim investigated in this work. This positive association could be spurious since confounders like the vector load are not available to be incorporated in the model. The disease-specific spatial field for G6PD deficiency is non-trivial with a small precision parameter *τ*_2_ ∈ (0.671; 2.131), even though based on the small weight parameter *ϕ*_2_ ∈ (0.014; 0.539), we note that the unstructured effect accounts for most of the variation in the disease-specific effect; this is also clear from figure 8, where we show the posterior means of the structured and unstructured effects for the countries under consideration. Concerning the shared spatial field, S, we illustrate S and cS for *α*_1_ = 0.2 and *α*_2_ = 0.8 in figure 8.

**Table 5.** Posterior inference for model (5.1) of the malaria and G6PD deficiency data.

	joint quantile model		joint quantile model		joint mean model	
	$\alpha_{1}=0.2,\alpha_{2}=0.8$		$\alpha_{1}=0.8,\alpha_{2}=0.2$			
parameter	mean	95% credible interval	mean	95% credible interval	mean	95% credible interval
*m*_1_	7.853	(6.572; 9.13)	7.887	(6.632; 9.138)	7.882	(7.245; 8.52)
*m*_2_	4.248	(3.746; 4.741)	4.151	(3.655; 4.639)	4.154	(3.739; 4.561)
*τ*_1_	309.46	(1.226; 2054.61)	11.31	(5.485; 24.16)	0.416	(0.236; 0.673)
*ϕ*_1_	0.352	(0.029; 0.868)	0.056	(0; 0.311)	0.189	(0.013; 0.611)
*τ*_2_	1.269	(0.671; 2.131)	131.5	(55.62; 326.2)	1.07	(0.564; 1.805)
*ϕ*_2_	0.168	(0.014; 0.539)	0.169	(0.015; 0.534)	0.196	(0.02; 0.582)
*τ*	0.104	(0.043; 0.218)	3.111	(1.182; 7.326)	107.9	(6.489; 438.352)
*d*	1.683	(0.378; 4.596)	1.704	(0.372; 4.784)	1.14	(0.08; 4.517)
*c*	0.214	(0.012; 0.442)	0.199	(−0.042; 0.444)	1.003	(0.384; 1.629)

On the contrary, for *α*_2_ = 0.2 and *α*_1_ = 0.8, the shared spatial field as well as the disease-specific spatial fields exhibit low spatial correlation since the precision parameters are estimated to be large, *τ* ∈ (1.182; 7.326), *τ*_1_ ∈ (5.485; 24.16), *τ*_2_ ∈ (55.62; 326.2). Also, the quantiles seem to be unrelated since *c* ∈ ( − 0.042; 0.444).

**Figure 9.**
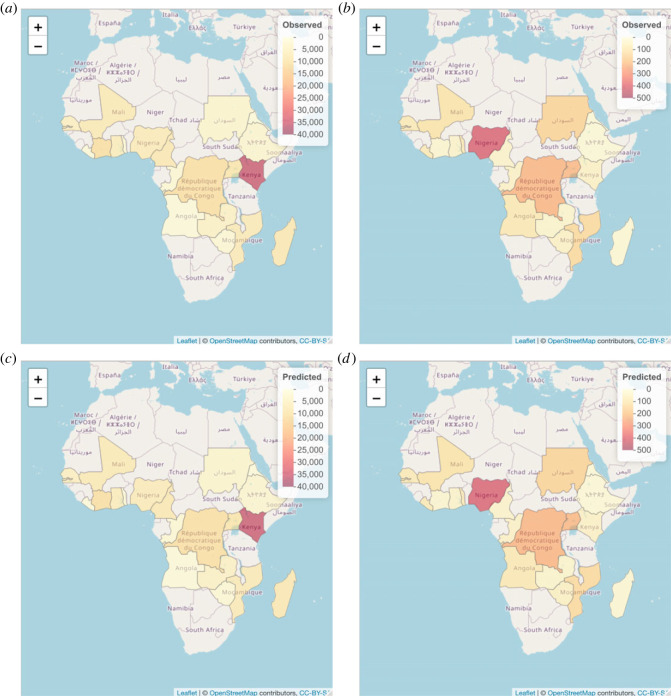
Observed (*a,b*) and predicted (*c,d*) cases for malaria (*a,c*) and G6PD deficiency (*b,d*) for model (5.1) with *α*_1_ = 0.2, *α*_2_ = 0.8.

We use the fitted joint quantile regression model and calculated in-sample predictions for each country and disease; these predictions are displayed in figure 9 together with the observed cases. We concur that the model seems to fit the data well.

To further investigate the model fit, we calculate the DIC and WAIC for the joint quantile models as well as the joint mean model; these results are presented in table 6. Note that for the data under consideration, model (5.1) with *α*_1_ = 0.2 and *α*_2_ = 0.8 is preferred based on the model selection criteria. This indicates some support for the hypotheses of high quantiles of G6PD deficiency being associated with low quantiles of malaria, even though here the association is counterintuitive.

**Table 6.** Model selection criteria for model (5.1) of the malaria and G6PD deficiency data; italics indicate the better fit.

	$\alpha_{1}=0.2,\alpha_{2}=0.8$		$\alpha_{1}=0.8,\alpha_{2}=0.2$	
model	DIC	WAIC	DIC	WAIC
model for $q_{i1,\alpha_{1}}$ only	168	164.8	168.4	166.4
model for $q_{i2,\alpha_{2}}$ only	246.8	241.4	246.2	240.3
sum of the separate models	414.8	406.2	414.6	406.7
joint quantile model	*413.6*	*402.2*	414.5	406.1
joint mean model	414.5	405.4	414.5	405.4

## 6. Concluding remarks

The motivation of this work stemmed from estimating the relative risk of malaria and G6PD deficiency, jointly, on the African continent. G6PD deficiency is considered to offer some resistance against malaria, based on anecdotal medical studies [5,9]. If this is the case, we would expect to see a lower incidence of malaria in areas where there are many cases of G6PD deficiency. As such, joint mean disease mapping will not provide the information needed to investigate these initial findings, since the hypothesis pertains to different quantile levels of the disease cases. Hence, we proposed a joint quantile disease mapping model of different quantiles for the joint inference of many diseases. We base the model on a model-based quantile regression approach that is shown to be more intuitive in the framework of disease mapping and also more robust to the phenomena of quantile crossing, without the joint modelling of multiple quantiles within the same disease. We use the efficient INLA framework to perform full Bayesian analysis of our proposed model.

Our main contribution is twofold. Firstly, we propose a very general joint quantile disease mapping model in which the correlation between different quantiles can be inferred and multiple diseases can be considered, together with an efficient computational framework for the inference thereof. Secondly, the significant correlation between a high quantile of G6PD cases and a low quantile of malaria cases encourages further investigation of this hypothesis based on the expanded data collection efforts as already underway at the Malaria Atlas Project. This analysis provides a statistical framework to investigate the anecdotal findings reported by medical professionals and could underpin future studies in this direction. The finding of a positive correlation between low quantile of malaria and a high quantile of G6PD is odd. We expected to find a negative correlation and this motivates further investigation based on more extensive data. Various shortcomings are evident as well, such as the absence of an indication of the vector load in the area. If there is an increased number of vectors in the area then the effect of the absence or presence of G6PD deficiency could be confounded, since the number of malaria cases will be high, even if half of the subjects have G6PD deficiency. If the vector load is low, however, then there will be few cases of malaria, regardless of whether G6PD deficiency is high or low. This extra information is needed to confidently affirm whether G6PD deficiency offers some resistance to malaria.

Future developments could include expanding the proposed model for geo-referenced data that are not aggregated by country but rather observed at individual locations. This extension could enable the application of a more accurate model for high resolution spatial data and could even be used to identify hotspots of either disease within a country to provide valuable information to public health officials. The computational framework we used, based on the INLA methodology, can be trivially extended to this case. Furthermore, we could incorporate temporal information into the model when these data become available, since model (4.1) can accommodate temporal structures as random effects; this may provide insights into the evolution of cases of the diseases over time.

## Data Availability

This article has no additional data.

## Appendix A. Model-based quantile regression for other discrete data-generating function

We show how the Binomial and the Negative Binomial distributions can be similarly extended to the continuous case. Their CDFs can be expressed as

$$Y^{*}∼\text{Binomial}(n,p)\;F_{Y^{*}}(y)=I_{1-p}(n-y,y+1)$$

and

$$Z^{*}∼\text{Negative Binomial}(r,p)\;F_{Z^{*}}(z)=I_{1-p}(r,z+1),$$

where *I*_*x*_(*a*, *b*) is the *regularized incomplete Beta function* defined as

$$I_{x}(a,b)=\frac{\text{B}(a,b,x)}{\text{B}(a,b)}\;\text{with}\;\text{B}(a,b,x)=\int_{x}^{1}t^{a}{\left(1-t\right)}^{b-1}\text{d}t.$$

The continuous extension is then obtained as

$$Y∼\text{Continuous Binomial}(n,p)\;F_{Y}(y)=I_{1-p}(n-y,y+1)$$

and

$$Z∼\text{Continuous Negative Binomial}(r,p)\;F_{Z}(z)=I_{1-p}(r,z+1).$$

These continuous extensions result in interpolation of both the cumulative distribution and quantile functions of the discrete counterparts. The advantage of this interpolation scheme is that the behaviour of the resulting continuous random variables mimics that of their discrete counterparts. In the discrete case, it is well known that the Poisson distribution is the limiting case of both the Binomial and the Negative Binomial and that the Binomial and Negative Binomial are entwined in a one-to-one relation. Theorem A.1 shows how these relationships between variables are preserved in the continuous case; hence, the two classes of distribution have similar meanings. From a modelling perspective, in fact, theorem A.1 justifies the interpretation (i) of the CP as an approximation for a Binomial-like distribution in the case of rare events, (ii) of the Continuous Negative Binomial as an over-dispersed version of the CP and (iii) of the Continuous Negative Binomial as the waiting time until the arrival of the *r*th success in a Binomial-like experiment.

Theorem A.1. *Let X be a Continuous Poisson random variable with parameter* *λ*, *Y* *be a Continuous Binomial with parameters* *n* *and* *p* *and* *Z* *be a Continuous Negative Binomial with parameters* *r* *and* *p*. *Then the following relations hold:*

(i) *for* *n* → ∞ *and* *p* → 0 *so that* *np* → *λ*
  A 1 $$F_{Y}(x)=\frac{\text{B}(x+1,N-x,p)}{\text{B}(x+1,N-x)}⟶\frac{\Gamma (x+1,\lambda )}{\Gamma (x+1)}=F_{X}(x),$$
(ii) *for* *r* → ∞ *and* *p* → 0 *so that* *rp* → *λ* *we have*
  $$F_{Z}(x)=\frac{\text{B}(x+1,r,p)}{\text{B}(x+1,r)}⟶\frac{\Gamma (x+1,\lambda )}{\Gamma (x+1)}=F_{X}(x),$$
(iii) *let* *W* *be a Continuous Binomial random variable with parameters* *s* + *r* *and* 1 − *p*, *then*
  $$F_{Z}(s)=1-F_{W}(r).$$

Proof.

(i) Immediately follows from Ilienko [50].
(ii) Follows trivially from (A 1).
(iii) Follows from
  $$\begin{array}{rl} F_{Z}(s) & =1-I_{p}(s+1,r)\\ & =1-I_{p}((s+r)-(r-1),(r-1)+1)\\ & =1-P(Y\leq r-1)\\ & =P(Y\geq r). \end{array}$$

 ▪

## Appendix B. Malaria and G6PD deficiency analysis

To investigate the relationship between other quantiles of malaria and G6PD deficiency, we consider the same model as in (5.1):

B 1 $$\begin{array}{rl} & y_{i1}|\lambda_{i1}∼\text{Poisson}\;(E_{i1}\lambda_{i1})\\ & y_{i2}|\lambda_{i2}∼\text{Poisson}\;(E_{i2}\lambda_{i2})\\ & \log(q_{i1,\alpha_{1}})=m_{1}+b_{i1}+S_{i}\\ \;\mathrm{and}\; & \log(q_{i2,\alpha_{2}})=m_{2}+b_{i2}+c\;S_{i}. \end{array}$$

We considered different values of *α*_1_ and *α*_2_ and the posterior inference of these models are presented in table 7.

For different values of *α*_1_ and *α*_2_, we observe that the correlation between the diseases are in opposite quantile levels since for the same quantile the large value of *τ* indicates that malaria and G6PD deficiency are uncorrelated through those quantiles, contrary to the findings in §5 for *α*_1_ = 0.2, *α*_2_ = 0.8.

**Table 7.** Posterior inference for model (B 1) of the malaria and G6PD deficiency data.

	*α*_1_ = 0.4, *α*_2_ = 0.8		*α*_1_ = 0.5, *α*_2_ = 0.5		*α*_1_ = 0.8, *α*_2_ = 0.8	
parameter	mean	95% credible interval	mean	95% credible interval	mean	95% credible interval
*m*_1_	7.87	(7.238; 8.504)	7.879	(7.252; 8.508)	7.907	(7.291; 8.524)
*m*_2_	4.255	(3.864; 4.637)	4.138	(3.726; 4.54)	4.255	(3.865; 4.636)
*τ*_1_	0.404	(0.228; 0.648)	0.409	(0.23; 0.656)	0.426	(0.24; 0.683)
*ϕ*_1_	0.183	(0.011; 0.606)	0.183	(0.011; 0.606)	0.182	(0.011; 0.611)
*τ*_2_	1.138	(0.602; 1.923)	1.020	(0.538; 1.720)	1.14	(0.599; 1.917)
*ϕ*_2_	0.195	(0.016; 0.603)	0.194	(0.016; 0.595)	0.189	(0.015; 0.591)
*τ*	1788.819	(121.382; 6657.269)	1804.623	(122.885; 6704.552)	1907.333	(137.889; 6953.007)
*d*	0.973	(0.062; 3.462)	0.939	(0.067; 3.436)	0.91	(0.063; 3.364)
*c*	1	(0.377; 1.623)	1.002	(0.375; 1.622)	0.998	(0.378; 1.625)
